# Pseudogout Diagnosed By Point-of-care Ultrasound

**DOI:** 10.5811/cpcem.2019.7.43244

**Published:** 2019-09-30

**Authors:** Anthony J. Halupa, Robert J. Strony, David H. Bulbin, Chadd K. Kraus

**Affiliations:** * Geisinger Medical Center, Department of Emergency Medicine, Danville, Pennsylvania; † Geisinger Medical Center, Department of Rheumatology, Danville, Pennsylvania

## Abstract

A 71-year-old male presented to the emergency department (ED) for worsening right knee pain for the prior 3–4 weeks. Point-of-care ultrasound (POCUS) of the right knee showed a pseudo-double contour sign. Subsequent ultrasound-guided arthrocentesis of the knee joint was performed, and fluid studies showed the presence of calcium pyrophosphate crystals, which was consistent with pseudogout. Ultrasound for detection of calcium pyrophosphate crystals in pseudogout and chondrocalcinosis has sensitivity of 86.7% and specificity of 96.4% making POCUS a valuable tool for diagnosing crystalline-induced arthropathy in the ED.

## CASE PRESENTATION

**Image 1:**
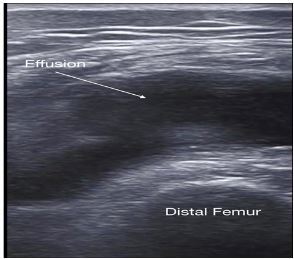
Short-axis view of the distal femur superior to the patella using a linear high-frequency transducer. A large knee joint effusion is shown anterior to the distal femur.

**Image 2:**
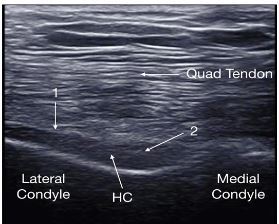
Short-axis view of the femoral condyle and hyaline cartilage (HC) using a high-frequency linear transducer. Mild hyperechoic enhancement of the superficial layer (arrow 1) and hyperechoic thickening within the intermediate layer of the HC (arrow 2) are visible-the so-called pseudo-double contour sign.

A 71-year-old male presented to the emergency department with worsening right knee pain and swelling for the prior 3–4 weeks. Past medical history was significant for gout treated with colchicine. The patient was afebrile. Physical exam demonstrated a swollen right knee, mild erythema, and limited range of motion. Point-of-care ultrasound (POCUS) of the right knee showed the findings depicted in Images 1 and 2. A POCUS-guided arthrocentesis was performed and confirmed the diagnosis suggested by POCUS.

## DISCUSSION

**Image 3:**
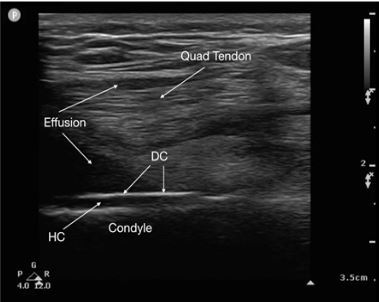
Short-axis view of the femoral condyle and hyaline cartilage (HC) using a high-frequency linear transducer, which shows intense hyperechoic enhancement of the superficial layer of the HC, the double contour (DC) sign. Thickening and hyperechoic enhancement of the intermediate layer is absent.

**Image 4:**
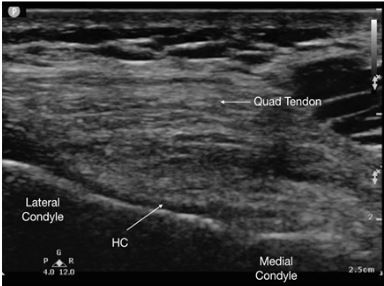
Short-axis view of the femoral condyle and hyaline cartilage (HC) of a normal knee using high-frequency linear transducer. Note the absence of hyperechoic enhancement of the superficial layer of the HC and absence of hyperechoic enhancement and thickening of the intermediate layer of the HC.

In this case, POCUS suggested the diagnosis of pseudogout by demonstrating the pseudo-double contour sign. The joint aspirate contained calcium pyrophosphate crystals, 161 white blood cells per cubic millimeter, no organisms on gram stain, and negative cultures. The pseudo-double contour sign is formed when calcium pyrophosphate crystals deposit in the hyaline cartilage^1^ (depicted in Image 2). This is opposed to the double contour sign seen in gout (Image 3), where monosodium urate crystals deposit on the surface of the articular cartilage causing hyperechoic enhancement of the superficial margin.^1^,^2^ Image 4 depicts a normal knee for comparison.

**CPC-EM Capsule**

What do we already know about this clinical entity?

*Ultrasound detection of calcium pyrophosphate crystals in pseudogout has high sensitivity and specificity per rheumatology literature*.

What is the major impact of the image(s)?

*These point-of-care ultrasound (POCUS) images will help emergency physicians diagnose and differentiate pseudogout and gout*.

How might this improve emergency medicine practice?

*Identifying pseudogout or gout on POCUS may decrease the need for aspiration and risk of infection in patients with previously diagnosed crystalline arthropathies*.

Ultrasound for detection of calcium pyrophosphate crystals in pseudogout and chondrocalcinosis has sensitivity of 86.7% and specificity of 96.4%.^3^ The double contour sign for gout has a reported sensitivity of 57% and 100% specificity for knees.^4^ POCUS is a valuable tool in diagnosing crystalline-induced arthropathy.^5^ Emergency physicians should consider using POCUS as an aid in diagnosing microcrystalline disease and guiding joint aspiration, as it may reduce the need for aspiration in the patient previously diagnosed with gout or pseudogout.
